# Hypotension Unresponsive to Fluid Resuscitation: A Case Report

**DOI:** 10.5811/cpcem.2022.4.55896

**Published:** 2022-07-27

**Authors:** Mahir Mameledzija, Erin Nasrallah, Molly Hartrich

**Affiliations:** * University of Illinois at Chicago, Department of Emergency Medicine, Chicago, Illinois; † Advocate Lutheran General Hospital, Department of Emergency Medicine, Park Ridge, Illinois

**Keywords:** pediculosis, lice infestation, hypotension, anemia, case report

## Abstract

**Introduction:**

Iron deficiency anemia is commonly seen in the emergency department (ED), and the cause can be complex and variable.

**Case Report:**

We present a case of a female without known medical history who presented to the ED for generalized weakness and was found to have severe anemia in the setting of chronic lice infestation.

**Conclusion:**

Severe and chronic pediculosis can cause chronic blood loss and be an unusual and rare cause of iron deficiency anemia. In the setting of anemia and hypotension unresponsive to fluid resuscitation, consideration should be given to early packed red blood cell transfusion and subsequent investigation of causes of severe anemia.

## INTRODUCTION

Anemia is a condition in which the number of red blood cells (RBC) in a patient’s blood are lower than normal and insufficient to meet an individual’s physiologic needs.^1^ While the etiology of a patient’s anemia may be complex and variable given the wide spectrum of conditions causing anemia, the initial workup and management of anemia is quite straightforward.^2^ Anemia is caused by bleeding, decreased production of RBCs, as in iron deficiency anemia (IDA), or increased destruction of RBCs, as in sickle cell anemia.

Lice infestation is a common finding among children in North America, with pediculosis capitis (head lice) known to be highly prevalent among the pediatric population.^3^ Pediculosis corporis (body lice) is rarer, and despite the prevalence of head lice infestation in the pediatric population, the literature on body lice infestation causing profound anemias is limited to a few case reports. In this case report, we present a patient with profound anemia likely due to chronic lice infestation.

## CASE REPORT

A 50-year-old woman with no known past medical history was brought into the emergency department (ED) by her older sister for evaluation of generalized weakness. Initial history provided by her sister suggested that the patient had not seen a doctor in years, had a history of “psychiatric problems,” and had been feeling weak for the prior month or two. The patient’s weakness and fatigue had extended to a point where her brother had to carry her around the house. The patient denied any medical problems and denied taking any prescription or over-the-counter medications.

On initial exam, the patient was hypotensive with a systolic blood pressure of 75 millimeters of mercury (mm Hg) and tachycardic to 110 beats per minute on arrival to the ED. The remainder of her vital signs were within normal limits. She had a normal mental status and was breathing comfortably on room air. At the time of evaluation the patient endorsed diffuse and profound weakness without focality for the prior month; the remainder of her review of systems was unremarkable. She reported she had been eating and drinking normally; however, family noted she would eat only dairy products and pudding.

On exam, she was a thin, ill-appearing person who was disheveled. Except for mild tachycardia, her cardiopulmonary exam was unremarkable. Her abdomen was benign. Her skin was hyperpigmented with multiple, scattered excoriations, and she had innumerable, wingless, dark-colored insects crawling in her hair and all over her body. One of the insects was isolated and inspected. It was noted to have a clear-tan shell with six legs and an elongated oval body, and was identified as a body louse.

While she was able to stand up straight and transfer herself to the exam bed from the wheelchair, according to the patient her weakness was too profound to attempt ambulation. On strength examination, she had diffuse, symmetric, upper and lower extremity weakness in the proximal to distal muscle groups. Apart from her profound weakness, there were no focal neurologic deficits, and the remainder of her exam was unremarkable.

Labs were collected, and an electrocardiogram (ECG) and radiograph were performed. For her hypotension, the patient was provided two liters of normal saline over one hour. Despite 30 cubic centimeters per kilogram of fluid resuscitation, her blood pressure and tachycardia persisted. Her labs were notable for lactic acid of 8.5 millimoles per liter (mmol/L) (reference range 0.5–2.2 mmol/L). There were no severe electrolyte abnormalities. Blood urea nitrogen/creatinine and liver function tests were within normal limits. We considered adrenal insufficiency with Addisonian crisis, especially given her exam finding of hyperpigmented skin, and a random cortisol level was added to her labs; however given normal sodium, potassium, and glucose level, steroids were deferred.

The patient’s hemoglobin and hematocrit resulted critically low at 1.6 grams per deciliter (g/dL) (reference range 12.0–15.5 g/dL) and 6.6% (36.0–46.5%), respectively (Table 1). Given concern for error, the complete blood count (CBC) was repeated with similar results. Her white blood cell count was 14.0 thousand cells per cubic microliter (K/μL) (4.2–11.0 K/μL) and platelets were 355 K/μL (140–450 K/μL). A rectal exam was performed without findings of gross blood or melena. Both the patient and her family denied trauma and gastrointestinal or genitourinary blood losses, and she had entered menopause approximately five years previously. Broad spectrum antibiotics were empirically started given concern for sepsis. The patient was subsequently resuscitated with two units of un-crossmatched packed red blood cells (PRBC) given the critical hemoglobin level, while an additional two units of crossmatched PRBCs were prepared in the blood bank.

The patient was quarantined and decontaminated in the ED exam room. Given her relative stability despite a critically low hemoglobin level, a traumatic etiology was thought to be very unlikely, and her hemoglobin had likely dropped slowly over a lengthy period. The patient was subsequently admitted to the medical intensive care unit for further evaluation and management of profound anemia and hypotension.

**CPC-EM Capsule**

What do we already know about this clinical entity?

*Iron deficiency anemia (IDA) has a wide spectrum of causes, many of which are not formally diagnosed during the patient’s stay in the Emergency Department*.

What makes this presentation of disease reportable?

*This case offers important considerations for patients unresponsive to initial crystalloid resuscitation and an uncommon etiology of profound anemia*.

What is the major learning point?

*Severe and chronic pediculosis can cause chronic blood loss, an unusual and rare cause of IDA*.

How might this improve emergency medicine practice?

*Early recognition offers the opportunity for point-of-care testing and earlier transfusion of packed red blood cells or other focused treatments*.

In total, the patient received four units of PRBCs in the ED and did not require further transfusions during her hospital course. Following the transfusion, her morning hemoglobin was 7.5 grams per deciliter (g/dL) (reference range 12.0–15.5 g/dL) and had a total of five stable hemoglobin levels on daily CBCs thereafter. She had an upper endoscopy and extensive laboratory workup led by hematology/oncology given the degree of her anemia; her subsequent workup did not reveal a source of bleeding, blood dyscrasia, or significant mineral or vitamin deficiency that would have contributed to her anemia. The patient responded very well to the PRBC transfusion, and after pediculosis was resolved her anemia did not return, which led us to believe her anemia was due to slow blood loss by chronic lice infestation.

## DISCUSSION

The patient’s labs were consistent with IDA. While it was unlikely the IDA was due to acute blood loss, the elevated reticulocyte count indicates that there was ongoing blood loss with appropriate bone marrow compensation. As noted by the family, the patient’s diet was exclusively dairy products; consumption of large amounts of cow’s milk may lead to IDA. This, however, is typically seen in children, and it is unlikely we would have seen such a profound anemia from heavy consumption of cow’s milk. Her lice infestation was impressive, and coupled with poor nutritional status, we concluded that her chronic and severe pediculosis was the most likely cause of her severe anemia. Although lice infestation is encountered with relative frequency in the ED, such a chronic and severe case leading to life-threatening anemia is quite rare. Spurred by this unique case, we reviewed the literature for case reports and studies of profound anemia due to pediculosis.

A review of the literature revealed case reports and one case series of patients with chronic and severe lice infections with profound anemias (Table 2). These were almost always microcytic IDA, with numerous cases requiring blood transfusion. Despite there being no causal or definite relationship between anemia and lice infestation, there is a theoretical relationship in that heavy infestation can lead to blood loss over the span of months and cause IDA, after other causes of IDA have been ruled out. Authors of a 2006 study published in the *International Journal of Dermatology* attempted to quantify the blood intake of a head louse in children.^4^ The authors concluded that heavy, chronic infestation would have a great potential to lead to iron deficiency and could be significant in an already iron-deficient child. In 2011 researchers performed a thorough chart review of ED cases and highlighted a possible association between lice infestation and profound IDA.^5^ While not pertinent to our patient, communicable diseases transmitted by lice such as epidemic typhus, trench fever, and epidemic relapsing fever are important considerations when treating patients with lice infestation as well as for medical staff safety. We found six individual case reports from 2015–2021 reporting lice infestation and associated anemia (Table 2).

Reports given by our patient’s family of her severe deconditioning and poor diet and hygiene suggested the possibility that her baseline hemoglobin was low and the infestation had worsened her anemia. It remained unclear how long she had been severely infested with lice and the trajectory of her weakness. Her hyperpigmented skin may have suggested adrenal insufficiency, particularly considering the initial refractory hypotension; however, her labs were not consistent with adrenal insufficiency and her inpatient workup did not reveal adrenal insufficiency. A skin condition associated with chronic body lice infestation, pediculosis corporis (also known as “vagabond’s disease”) demonstrates thickened and darkened skin similar to that of our patient.

## CONCLUSION

Despite the limited literature on the hematologic consequences of chronic lice infestation, this case offers important considerations for unknown etiologies of profound anemia and also for patients unresponsive to initial crystalloid resuscitation. In cases where a patient may be severely ill and there is lag time for labs to result, this may offer the opportunity for point-of-care testing and allow for earlier transfusion of packed red blood cells or other focused treatments. To our knowledge, the vast majority of the literature discussing severe iron deficiency anemia due to pediculosis has been in reference to IDA in young children and animals. This case report illustrates an important cause of IDA in older adults, especially those who are vulnerable and do not regularly interact with the medical community.

## Figures and Tables

**Table 1:** Lab results in adult patient with severe body lice infestation.

Lab result	Value	Reference range
White blood cell	14.0 K/mcL	4.2–11.0 K/mcL
Red blood cell	1.0 million/mm3	4.00–5.20 million/mm3
Hemoglobin	1.6 g/dL	12.0–15.5 g/dL
Hematocrit	6.60%	36.0–46.5%
Mean corpuscular volume	66 fL	78.0–100.0 fL
Mean corpuscular hemoglobin	16 pg	26.0–34.0 pg
Mean corpuscular hemoglobin concentration	24.2 g/L	32.0–36.5 g/L
Red cell distribution width	21.90%	11–15.0%
Platelets	355 K/mcL	140–450 K/mcL
Iron	14 μg/dL	50–170 μg/dL
Iron percent saturation	2%	15%–45%
Total iron binding capacity	496 μg/dL	250–450 μg/dL
Ferritin	4 ng/mL	8–252 ng/mL
Lactate dehydrogenase	346 U/L	82–240 U/L
Reticulocyte count	3.60%	0.3–2.5%

*K/mcL*, thousand cells per microliter; *million/mm3*, million per cubic millimeter; *g/dL*, grams per deciliter; *fL*, femtoliters; *pg*, picograms; *g/dL*, gram per deciliter; *μg/dL*, microgram per deciliter; *ng/mL*, nanogram per milliliter; *U/L*, units per liter.

**Table 2:** Previously reported cases of pediculosis and anemia.

Author	Patient	Type of infestation	HgB level
Hau and Muhi-Iddin.^6^	11-year-old female	Pediculosis Capitis	4.2 g/dL
Althomali, Alzubaidi and Alkhaldi.^7^	23-year-old female	Pediculosis Capitis	2.2 g/dL
Ronsley et al.^8^	4-year-old male	Pediculosis Capitis + Corporis	2.2 g/dL
Woodruff and Chang.^9^	74-year-old female	Pediculosis Capitis + Corporis	3.8 g/dL
Batool et al.^10^	32-year-old male	Pediculosis Capitis + Corporis	6.3 g/dL
Fustino, Waddell and Panzer.^11^	12-year-old female	Pediculosis Capitis + Corporis	4.7 g/dL

*HgB*, hemoglobin; *g/dL*, gram per deciliter.
